# Report of a New Sand Fly (Diptera: Psychodidae) Species, *Sergentomyia* (*Neophlebotomus*) *pradeepii* n. sp. from Madhya Pradesh, India

**DOI:** 10.3390/insects16060598

**Published:** 2025-06-06

**Authors:** Harish Kumar Shah, Pananchikkaparambil Abdu Fathima, Manju Rahi, Prasanta Saini

**Affiliations:** ICMR–Vector Control Research Centre, Puducherry 605006, India; harish.kumar883@gmail.com (H.K.S.); fathimapa547@gmail.com (P.A.F.); drmanjurahi@gmail.com (M.R.)

**Keywords:** Phlebotomine sand flies, *Sergentomyia* (*Neophlebotomus*) *pradeepii*, COI barcode, Madhya Pradesh, India

## Abstract

Madhya Pradesh, a biodiversity-rich state in central India, reports sporadic non-indigenous leishmaniasis cases. Systematic entomological surveillance as part of molecular xenomonitoring in sand flies led to the discovery of a new species, *Sergentomyia (Neophlebotomus*) *pradeepii* n. sp. (Diptera: Psychodidae), from Johariya village in Sagar district, Madhya Pradesh, India. A systematic cross-sectional survey of sand flies was conducted in Bhopal, Sagar, and Hoshangabad districts of Madhya Pradesh. Standard collection methods were employed for two months, i.e., from July to August 2023. DNA barcoding targeting the mitochondrial Cytochrome c oxidase subunit I (COI) gene was performed, and the generated sequences were phylogenetically analyzed. *Se.* (*Neo.*) *pradeepii*, a newly recorded sand fly species, is reported in this study. Its taxonomic relationship to other congeners of subgenus *Neophlebotomus* is discussed. COI barcoding and phylogenetic analysis established that the specimens fit into the same taxonomic group, exhibiting negligible gene flow within the population, while a 13.4% genetic distance from congeners establishes it as a separate species. Madhya Pradesh, with its rich biodiversity and favorable conditions for sand fly proliferation, lacks systematic entomological surveillance. This study enhances the knowledge of the state’s sand fly fauna by reporting and providing a detailed morphological and molecular description of the new species.

## Simple Summary

A systematic entomological survey in Madhya Pradesh, India, led to the discovery of a new sand fly species, *Sergentomyia* (*Neophlebotomus*) *pradeepii* n. sp., from Johariya village in the Sagar district. Sand flies were collected from Bhopal, Sagar, and Hoshangabad districts between July and August 2023. DNA barcoding of the mitochondrial COI gene and phylogenetic analysis confirmed the new species, showing a 13.4% genetic distance from related species and minimal gene flow within the population. This study provides important morphological and molecular data, contributing to the understanding of sand fly biodiversity in Madhya Pradesh, a region lacking systematic surveillance.

## 1. Introduction

Leishmaniasis, classified as a neglected tropical disease (NTD), is caused by protozoan parasites belonging to the genus *Leishmania* (William Leishman, 1901). This disease has been endemic for centuries in nations including Bangladesh, Brazil, Ethiopia, India, Nepal and Sudan [1]. Phlebotomine sand flies (Diptera: Psychodidae) act as vectors for *Bartonella* and certain arboviruses (Chandipura virus, Toscana virus, etc.), along with *Leishmania,* designating it of significant medical importance [2]. Recently, a comprehensive global review of these sand flies, identifying 1060 species distributed across the old and new world, was documented by Galati and Rodrigues (2023) [3]. In India, 69 species of sand flies have been documented, with the recent addition of 2 newly identified species from the Western Ghats region of Kerala making a total of 71 species [4,5,6].

Madhya Pradesh, the second largest state in India with a geographic area of 308,000 km^2^, lies in the north-central part of the country. Its boundaries are demarcated by the Ganga-Yamuna plains to the north, the Aravalli range to the west, the Chhattisgarh plain to the east, and the Tapti valley and Maharashtra plateau to the south. The state’s landscape is primarily defined by the Narmada-Sone Valley, which stretches east to west across its expanse [7]. Madhya Pradesh is rich in biodiversity, with 31% of its area under forest cover, encompassing numerous national parks, wildlife sanctuaries, biosphere reserves, and tiger habitats [8]. Moreover, the state is home to diverse ethnic groups, with Scheduled Castes (SCs) and Scheduled Tribes (STs) constituting 15.6% and 21.1% of the population, respectively. Agriculture remains the primary livelihood for a majority of the population [9].

Unlike the eastern states of India, which are endemic for visceral leishmaniasis (VL or kala-azar), or emerging endemic regions like Kerala in the south and Himachal Pradesh in the north, Madhya Pradesh reports only sporadic cases. States such as Assam, Gujarat, Jammu and Kashmir, Haryana, Puducherry, Sikkim, Tamil Nadu, and Uttaranchal exhibit similar trends [1,10,11]. The last recorded case of VL in Madhya Pradesh, reported in 2011, was attributed to migration from the endemic state of Bihar (VL and Post Kala-azar Dermal Leishmaniasis [PKLD] endemic region) [12]. According to Shah et al. (2023) [4], only 11 sand fly species belonging to three genera *Grassomyia* (Theodor, 1948) (1 species), *Phlebotomus* (Rondani and Berté, 1840) (4 species), and *Sergentomyia* (França and Parrot, 1919) (6 species) have been documented in Madhya Pradesh to date.

Shah et al. (2024) [13] conducted a cross-sectional entomological survey focusing on molecular xenomonitoring of leishmaniasis across three districts of Madhya Pradesh. This former study resulted in the identification of a new sand fly species, *Sergentomyia* (*Neophlebotomus*) *pradeepii* n. sp. Detailed morphological and molecular depictions of this species are presented in this article.

## 2. Materials and Methods

### 2.1. Study Area

**Figure 1:**
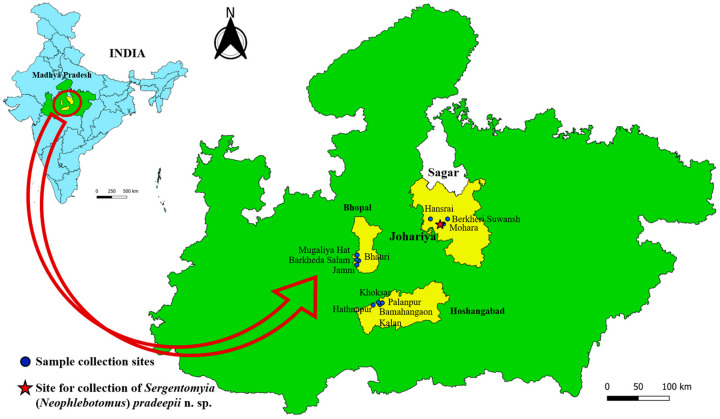
Sand fly specimen collection area in Johariya village of Sagar district in Madhya Pradesh, India.

A cross-sectional entomological collection of sand flies was conducted from a total of twelve sites situated in three different districts of Madhya Pradesh state, i.e., Bhopal, Sagar and Hoshangabad, for the period of July–August 2023 (Figure 1).

Bhopal district is highly urbanized, with most residents in the city and a small tribal population (Gonds and Bhils) on its outskirts. Sagar is predominantly rural, with agriculture as the primary livelihood and a moderate tribal population, mainly Gond and Korku tribes, engaged in farming and forest-related activities. It is also a regional hub for education and culture. Hoshangabad, located along the Narmada River, features rural and semi-urban settlements. Its economy relies on agriculture, forestry, and tourism linked to the river and jungle safaris. The Gond and Korku tribes, integral to the district’s traditions, depend on agriculture and forest resources (https://mpdistricts.nic.in/ (accessed on 22 April 2025)).

The population in these districts resides in varied housing conditions. Urban areas have relatively developed infrastructure with planned residential colonies and slum pockets, where housing is congested with limited sanitation and water access. In rural areas, housing is mostly kutcha (temporary) or semi-pucca (partially permanent), with mud walls and thatched or tiled roofs, often lacking basic amenities, especially in tribal and remote regions. Tribal communities predominantly live in kutcha houses made of mud, bamboo, or thatch, with rudimentary conditions in forested villages. However, government initiatives in recent years have improved housing and basic facilities, particularly for economically weaker and rural/tribal populations.

During July and August, the state experiences heavy rainfall, high humidity (over 70%), and temperatures ranging from 24 °C to 30 °C, creating ideal conditions for sand fly proliferation. A cross-sectional sand fly entomological collection was conducted during this period using standard entomological methods, including light traps, mechanical aspirators, and sticky traps [14]. Resting collections with mechanical aspirators were performed from 9:00 am to 12:00 noon, while light trap collections occurred from 6:00 pm to 8:00 am, covering evening and night hours. As this was a cross-sectional survey, sample collection was conducted only once at each site, with two trap collections per site, resulting in a total of 24 trap collections during this period.

### 2.2. Morphological Identification of Sand Flies

Sand fly specimens collected from various sites were transported to the Indian Council of Medical Research Vector Control Research Centre, Field Station in Kottayam, Kerala, India and preserved in 70% ethanol. Dissections were performed using a stereomicroscope (Weswox Optik-SZM-100, The Western Electric and Scientific Works, Ambala Cantt, India), and specimens were mounted on microscopic slides in Hoyer’s medium. Species-level identification was carried out using a binocular microscope (Primostar 3, Carl Zeiss Suzhou Co., Ltd., Shanghai, China) with reference to standard taxonomic keys [15,16]. Certain specimens, both male and female, exhibited features distinct from those described in existing taxonomic keys and literature. These unique taxonomic traits distinguished them from other species within the *Sergentomyia* subgenus *Neophlebotomus* (França and Parrot, 1920), such as *Se.* (*Neo.*) *ashwanii* (Saini et al., 2024), *Se.* (*Neo.*) *gemmea* (Lewis & Jeffery, 1978), *Se. (Neo.*) *iyengari* (Sinton, 1933), and *Se.* (*Neo.*) *monticola* (Srinivasan, Jambulingam & Kumar, 2014) [6,16,17].

Morphological investigation of the suspected novel species was performed using binocular microscope equipped with a micrometer, with all measurements recorded in micrometers (µm). Images highlighting key distinguishing features were captured using a microscope equipped with camera. For morphological evaluation, a holotype female and an allotype male were used alongside paratype specimens. Terminologies for species description followed those outlined by Galati et al. (2017) [18] and nomenclature adhered to the guidelines of the International Code of Zoological Nomenclature (ICZN) [19]. Measurements (in µm) for the holotype female and allotype male of *Se.* (*Neo.*) *pradeepii* n. sp. are detailed in Table 1.

### 2.3. Molecular Identification

Whole genomic DNA (gDNA) was extracted from the legs of individual sand flies using the commercially available QIAmp DNeasy Kit (Qiagen, Hilden, Germany), following the manufacturer’s protocol. The specimens were homogenized using a motor and pellet, and the final DNA was eluted in 30 μL of nuclease-free molecular-grade water. DNA barcoding was conducted from sand fly samples, targeting the mitochondrial Cytochrome c oxidase subunit I (COI) gene (~720 bp). Amplification of the gene followed the method described by Kumar et al. (2012) [20]. Bidirectional sequencing of the gene of interest was performed using the same primers. The nucleotide sequences obtained were further submitted in the National Center for Biotechnology Information (NCBI) GenBank for reference and accessibility.

### 2.4. Phylogenetic Analyses

Phylogenetic analysis was conducted by comparing sequences with GenBank using BLAST and aligning them in MEGA 11.0. The phylogenetic tree was constructed using the Neighbor-Joining method with the Kimura 2.0 parameter model and 1000 bootstrap replicates. Genetic distance and related parameters were also calculated using MEGA 11.0 to provide further insights into evolutionary relationships.

## 3. Results

Family: Psychodidae Newman, 1834.

Subfamily: Phlebotominae Rondani & Berté, in Rondani 1840.

Genus: *Sergentomyia* França and Parrot, 1920.

Subgenus: *Neophlebotomus* França and Parrot, 1920.

**Figure 2:**
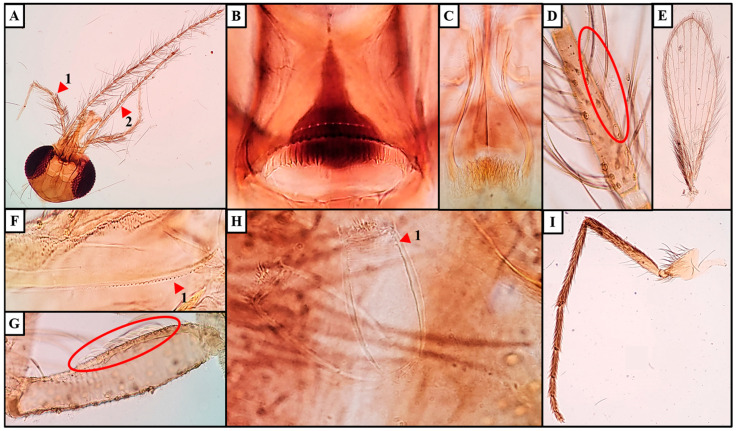
*Sergentomyia* (*Neophlebotomus*) *pradeepii* n. sp. (Female) (**A**–**I**): (**A**) dissected head with 1. palps and 2. flagellomere; (**B**) cibarium with horizontal cibarial teeth and pigment patch; (**C**) pharynx; (**D**) f2 with ascoid; (**E**) wing; (**F**) 1. maxillary teeth (external and internal); (**G**) Newstead’s scales on p3; (**H**) 1. spermatheca with faint striations at the apical end; (**I**) dissected fore leg.

**Figure 3:**
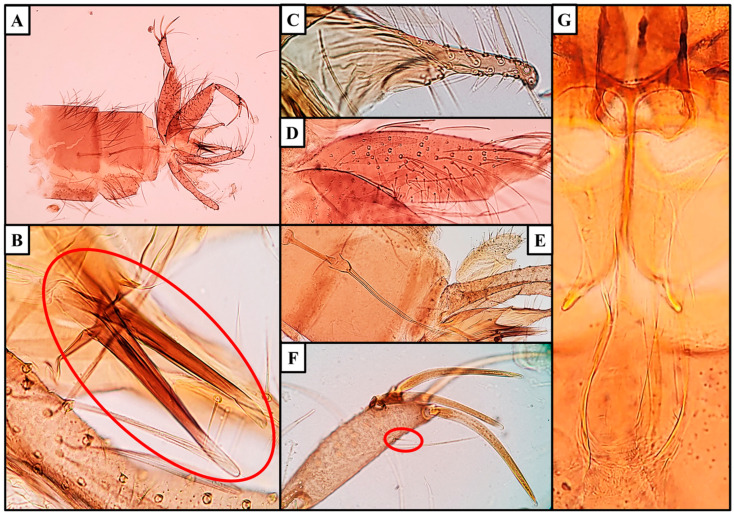
*Sergentomyia* (*Neophlebotomus*) *pradeepii* n. sp. (Male) (**A**–**G**): (**A**) dissected terminalia; (**B**) aedeagus/parameral sheath; (**C**) paramere with setae; (**D**) gonocoxite with setae on median tuft; (**E**) sperm/genital pump, filament/aedeagal duct and aedeagus/parameral sheath; (**F**) gonostylus with spines along with accessory setae; (**G**) cibarium and pharynx.

Species: *Sergentomyia* (*Neophlebotomus*) *pradeepii* n. sp. Shah et al. (Figure 2 and Figure 3).

### 3.1. Female

*Holotype female*: specimen exhibits a dark golden-brown coloration. Head measures 412 µm in length and 465 µm in width, with an interocular distance of 172 µm and a labrum length of 245 µm. Hypopharynx is almost smooth, featuring rudimentary teeth on each side. Maxilla has 3–5 internal teeth and 40–44 external teeth. The palpal formula is 5,4,3,2,1 (p5 > p4 > p3 > p2 > p1) and the palpomere measurements are as follows: p1 = 101 µm, p2 = 149 µm, p3 = 153 µm, p4 = 163 µm, and p5 = 338 µm. About 40 club-like Newstead’s sensilla are located on the middle of p3, while no similar structures are observed on other palpal segments. One distal spiniform seta is present on p3, five on p4, and nineteen on p5. Antennal segments measure f1 = 231 µm, f2 = 110 µm, and f3 = 109 µm (f1 > f2 + f3). Each antennal segment (f1–f13) has a pair of ascoids extending almost to the next segment, with an ascoid length on f2 measuring 59 µm. Simple setae (SS) are absent on f1–f13 and are reported as 18-24 on f14. A single distal papilla is observed on f1–f2, absent on f3–f10, two are reported on f11, four on f12, two on f13, and five on f14.

*Cibarium*: a ventral plate with approximately 11 distinct denticles or fore-teeth and a dark, pear-shaped pigment patch with a pointed tip on the dorsal plate. It contains 29–31 distinct, pointed horizontal teeth arranged in a tapering row. Pharynx is pot-shaped with a broader base, heavily armed with a group of long spicules or teeth, measuring 155 µm in length and 61 µm in width, with a pharyngeal armature depth of 39 µm. Pharynx is approximately 1.6 times longer than its width.

*Wings*: 1660 µm in length and 568 µm in width, with the principal vein sections measuring alpha = 519 µm, beta = 302 µm, gamma = 297 µm, delta (R1 overlap) = 173 µm, and R5 = 1345 µm. The wing index (alpha/beta) is 1.51.

*Fore leg*: coxa 280 µm, trochanter 80 µm, femur 748 µm, tibia 715 µm, tarsomeres T1 328 µm, T2 203 µm, T3 133 µm, T4 113 µm, and T5 98 µm.

*Genitalia*: cylindrical spermathecae with a short, cylindrical terminal knob. Each spermatheca is thick-walled and displays faint internal wrinkles or striations only at the tip, without distinct segmentation throughout its length. Spermatheca measures 61 µm in length and 29 µm in width and features secretory cells at the apical end. Individual spermathecal duct is short, sometimes not visible, with faint striations, while the common spermathecal duct is not visible. Cerci are simple and measure 157 µm in length. Genital furca is not clearly distinguishable (Figure 2).

### 3.2. Male

*Allotype male*: specimen exhibits a dark golden-brown coloration, consistent with the female specimen. Head measures 382 µm in length and 419 µm in width, with an interocular distance of 182 µm and a labrum length of 196 µm. Teeth on the hypopharynx and maxilla are rudimentary. Palpal formula is identical to the female (5,4,3,2,1: p5 > p4 > p3 > p2 > p1), with the following palpomere measurements: p1 = 85 µm, p2 = 127 µm, p3 = 150 µm, p4 = 165 µm, and p5 = 317 µm. Approximately 38 club-like Newstead’s sensilla are observed on the middle of p3, but absent on other palpal segments. One distal spiniform seta is present on p3, four on p4, and nineteen on p5. Antennal segments measure f1 = 261 µm, f2 = 123 µm, and f3 = 122 µm, (f1 > f2 + f3). Each antennal segment (f1–f13) has a pair of ascoids extending almost to the next segment, with an ascoid length on f2 of 52 µm. Simple setae (SS) are absent on f1–f13 and are reported as 15–20 on f14. A single distal papilla is observed on f1–f2, absent on f3–f10, two are reported on f11, three on f12, two on f13, and four on f14.

*Cibarium*: 23–27 rudimentary and faint horizontal teeth on the ventral plate, with 6–9 indistinct denticles or fore-teeth. Pigment patch is present on the dorsal plate but is less distinct than in the female specimen. Pharynx is pot-shaped with a marginally broader base, lightly armed with spicules or teeth compared to the female. Length is 165 µm, width is 54 µm and pharyngeal armature depth is 29 µm. Pharynx is about 3.1 times longer than its width.

*Wings*: length 1570 µm and width 508 µm. Length of principal vein sections: alpha 438 µm, beta 240 µm, gamma 270 µm, delta (R1 overlap) 169 µm and R5 length 1188 µm. Wing index (alpha/beta) is 1.82.

*Fore leg*: coxa 283 µm, trochanter 75 µm, femur 680 µm, tibia 705 µm and tarsomeres T1 300 µm, T2 188 µm, T3 128 µm, T4 108 µm, and T5 83 µm.

*Genitalia*: length of sperm pump is 107 µm, length of aedeagal duct is 310 µm, length of sperm pump + length of aedeagal duct is 417 µm and ratio of length of sperm pump/length of aedeagal duct is 2.9, with the aedeagal duct being much longer. Aedeagal filament is straight, striated, and slender throughout. Ejaculatory apodeme is 85 µm long. Gonocoxite is 272 µm in length and features a median tuft of 55–60 internal setae. Gonostyle is 119 µm long and has four thick spines: two apical and two subapical or subterminal, with a tapered end, measuring an average of 86 µm. The distance between the apical and subapical spines is 23 µm. An accessory spine is located just below the subapical spine. The paramere is hooked, measuring 176 µm, and has about 30 strong upward-facing setae. The parameral sheath or aedeagus is a tapered shape with a blunt end. Epandrial lobes are 225 µm long (Figure 3).

### 3.3. Diagnosis of Sergentomyia (Neophlebotomus) pradeepii n. sp.

The features of horizontal/recumbent hairs on abdominal tergites (2–6) found in both sexes, cibarial teeth in a row, fore teeth sometimes present and usually upwards pointing, pigment patch usually present and style of male with four prominent spines and an accessory seta are explicit to the genus *Sergentomyia*. Cibarial teeth are parallel, often equal but not very narrow. The pharynx is slender, with teeth or scales, or nearly unarmed. Antenna I is longer than II + III. Spermathecae are thin-walled capsules and with faint striations in females. The aedeagus is slender with a rounded tip and a hooked paramere in males. These common and identifying taxonomic characters confirm the inclusion of the species into the subgenera *Neophlebotomus* of genera *Sergentomyia*. Cibarium has a row of 27–30 hind/cibarial teeth in both sexes (rudimentary in males). In females, cibarial teeth are arranged in a row narrowing to a fine point. In males, teeth are rudimentary. In addition to the cibarial teeth, both sexes have 9–11 fore-teeth or small denticles (very much distinct in females as compared to males). These features are characteristic in *Sergentomyia* (*Neophlebotomus*) *pradeepii* n. sp.

### 3.4. Morphological and Molecular Variability

**Figure 4:**
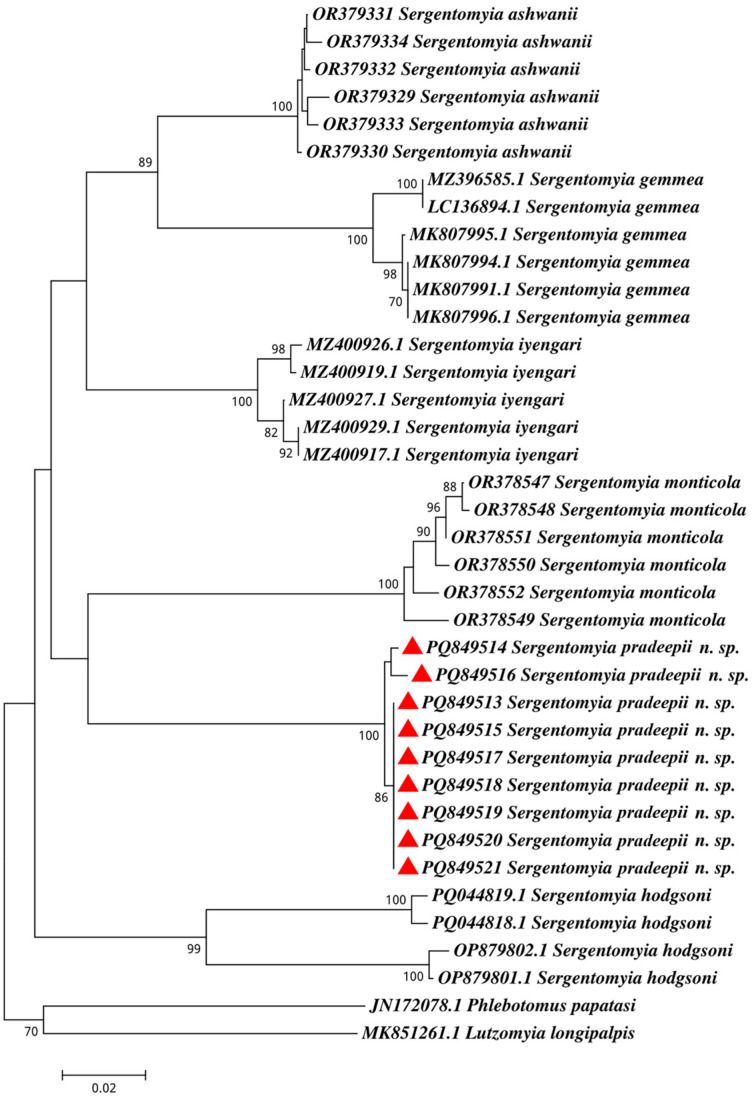
Phylogenetic analysis of mitochondrial Cytochrome *c* oxidase subunit I (COI) gene sequences for species of *Sergentomyia* (*Neophlebotomus*) *pradeepii* n. sp. (marked with red triangles) along with *Se.* (*Neo.*) *ashwanii, gemmea, iyengari*, *monticola* and *hodgsoni*; outgroup; *Ph. papatasi* and *Lutzomyia longipalpis*.

The unique morphological identifying features of the holotype, paratype female, allotype, and paratype male were consistent (Table 1). All specimens of *Se.* (*Neo.*) *pradeepii* n. sp. were collected from the same habitat type (cattle sheds and adjacent human dwellings) and district (Sagar, Madhya Pradesh, India), showing similar taxonomic features. DNA barcode sequences from all the specimens exhibited only six nucleotide variations and a negligible genetic distance (K2P), indicating they belong to a single taxonomic group. In contrast, the overall genetic distance between *Se.* (*Neo.*) *pradeepii* n. sp. and closely related congeners was 13.4%. *Sergentomyia ashwanii* was reported as 14.8%*, Se. gemmea* as 16.9%, *Se. iyengari* as 13.8%, *Se. monticola* as 16.1%, and *Se. hodgsoni* as 18.3% of GD with *Se. pradeepii* (Figure 4).

### 3.5. Type Locality and Materials

*Se.* (*Neo.*) *pradeepii* constitutes about 10% (28 specimens) of the total species composition in Johariya village. The type locality for these specimens (both paratype female and male) includes cattle sheds and adjacent rooms in Johariya village, tehsil Sagar, gram panchayat Masurhai, district Sagar, Madhya Pradesh, India (GPS coordinates: 23.78° N, 78.52° E, altitude: 534 m above sea level) on 24 and 25 July 2023 in light trap (evening biting collection) and mechanical aspirator (day resting collection). Voucher specimens of the holotype female and allotype male were mounted on glass slides, provided with a unique code along with all collection details and were deposited at the ICMR-Vector Control Research Centre (VCRC) museum, Puducherry-605006, India. Paratype specimens were submitted to the National Museum, Zoological Survey of India (ZSI), Alipur, Kolkata, India. Molecular analysis samples were submitted to the National Center for Biotechnology Information (NCBI) GenBank under accession numbers PQ849513-PQ849521. The type specimens include the female holotype (voucher GD-2960[VCRC]) and male allotype (voucher GD-2459[VCRC]), both deposited at the ICMR-VCRC museum, Puducherry, India.

### 3.6. Etymology

The new species *Se.* (*Neo.*) *pradeepii* is named in honor of Dr. N. Pradeep Kumar, Former Scientist (Director Grade), Indian Council of Medical Research—Vector Control Research Centre, Field Station, Kottayam, Kerala, in recognition and gratitude of his contributions to public health entomology, with a special emphasis on his pioneering work in insect molecular taxonomy.

### 3.7. ZooBank Registration

In accordance with Section 8.5 of the amended 2012 version of the ICZN [19], the details of the new species have been submitted to ZooBank. The associated Life Science Identifiers (LSIDs) are as follows: urn:lsid:zoobank.org:pub:032111CF-6707-47FF-A7AB-5CEBBC82C5D6 and urn:lsid:zoobank.org:act:D68A233A-5F6B-4124-B102-EBF366B1FAA5.

## 4. Discussion

A comprehensive updated checklist of sand flies in Madhya Pradesh, central India, was documented by Shah et al. (2023) [4]. The current entomological surveillance study, part of molecular xenomonitoring of leishmaniasis across India, focused on three districts: Bhopal, Sagar, and Hoshangabad, due to reported leishmaniasis cases [12,13]. These districts provide ideal conditions for sand fly proliferation during pre-monsoon (July–August), including high humidity, rainfall, vegetation, and diverse blood-feeding hosts. This study presents the morphological and molecular description of a newly identified sand fly species, *Se.* (*Neo.*) *pradeepii* n. sp., from Johariya village in Sagar district.

The genus *Sergentomyia* encompasses several sand fly species categorized into various subgenera, including *Neophlebotomus*, one of the most diverse with over 20 species recorded from the Oriental/Indomalayan region [10,15,16]. According to published literature, 19 *Sergentomyia* species have been documented in India to date [4]. These include *Se.* (*Neo.*) *arboris* (Sinton, 1931), *ashwanii* (Saini et al., 2024), *chakravarti* (Mitra, 1953), *dhandai* (Lewis, 1978), *gemmea* (Lewis and Jeffery, 1979), *hodgsoni hodgsoni* (Artemiev, 1976), *iyengari* (Sinton, 1932), *kottamala* (Kaul, 1993), *kurandamallai* (Kaul, 1993), *linearis* (Lewis, 1978), *malabarica* (Annandale, 1910), *monticola* (Srinivasan et al., 2014), *nilamburensis* (Kaul and Prabha, 1993), *perturbans* (de Meijere, 1967), *purii* (Sinton, 1931), *quatei* (Lewis, 1978), *verghesei* (Kaul, 1993), and *zeylanica* (Annandale, 1910) [4,5].

While referring to standard taxonomic keys, *Se.* (*Neo.*) *pradeepii* n. sp. specimens exhibited similarities to congeners under the genera *Sergentomyia* and subgenera *Neophlebotomus,* which includes *Se.* (*Neo.*) *ashwanii*, *dhandai*, *gemmea*, *iyengari*, *monticola*, and *hodgsoni* [5,15,16]. However, distinct morphological traits set them apart, leading to their classification as a new species. A female of *Se. ashwanii* has 10–12 cibarial teeth, 4–6 denticles, a funnel-shaped pigment patch, and thin-walled spermatheca with striations. *Se. dhandai* has 24 pointed cibarial teeth, a broad pigment patch with a dark cuticle at the teeth base, and thick-walled spermatheca with faint wrinkles and a short cylindrical knob. *Se. gemmea* features 10 tapering cibarial teeth, a pale pigment patch, and a narrow spermatheca with proximal wrinkles and a knob in a deep pit. *Se. iyengari* has 20 cibarial teeth, central teeth smaller than others, and a thick, forward-projecting pigment patch. *Se. monticola* has 10 cibarial teeth, 3–4 ventral plate denticles, and a golden-brown dome-shaped pigment patch. *Se. hodgsoni* has 50–60 cibarial teeth [5,15,16]. In contrast, *Se. pradeepii* females possess 29–31 distinct tapering cibarial teeth, approximately 11 denticles or fore-teeth, and a dark, pear-shaped pigment patch with a pointed tip. Spermathecae are cylindrical, thick-walled with faint wrinkles or striations, and feature a short terminal knob, which distinctly differentiates *Se.* (*Neo.*) *pradeepii* from its congeners.

Males of these congeners also differ from *Se. pradeepii* n. sp. A male of *Se. ashwanii* has an irregular arrangement of 10–12 cibarial teeth and an indistinct funnel-shaped pigment patch. *Se. dhandai* has about 24 pointed cibarial teeth on a deep arc without a pigment patch. *Se. gemmea* has a cibarium with approximately 6 irregular hind teeth, about 20 fore-teeth, and an indefinite pigment patch. Male *Se. monticola* displays about 10 parallel but irregular hind teeth with indistinct denticles in the cibarium’s ventral plate. *Se. iyengari* features a cibarium with conspicuous fore teeth, while *Se. hodgsoni* differs by having a spinose process at the base or on the ventral part of the paramere [5,15,16]. In contrast, *Se. pradeepii* males exhibit 23–27 rudimentary, faint horizontal teeth on the ventral plate, along with 6–9 indistinct denticles or fore-teeth, and a less distinct pigment patch compared to females. All congener species, including *Se. pradeepii*, share a similar arrangement of spines on the gonostyle of the male genitalia, though variations exist in the position and distance of the accessory seta relative to the spines.

Molecular taxonomy through DNA barcoding and phylogenetic analysis established the association within *Se. pradeepii* n. sp. specimens, showing minimal genetic distance (GD) and six nucleotide variations. The overall GD with congener species was 13.4%, with *Se. ashwanii*, *gemmea*, *iyengari*, *monticola*, and *hodgsoni* having GDs of 14.8%, 16.9%, 13.8%, 16.1%, and 18.3%, respectively, from *Se. pradeepii*. Population genetic analysis using MEGA 11.0 indicated high genetic diversity (*H_ST_* = 0.852) and negligible gene flow (*N_m_* = 0.003). These taxonomic differences and molecular findings suggest that *Se.* (*Neo.*) *pradeepii* n. sp. is distinct and divergent from other previously described species under the subgenera of *Neophlebotomus*. 

## 5. Conclusions

This study presents a detailed morphological and molecular description of *Se.* (*Neo.*) *pradeepii* n. sp., a newly recorded sand fly species from Madhya Pradesh, India. Previously, the state had records of only 11 sand fly species, which has now been updated to 12 following this report. Systematic entomological surveillance is essential for understanding the region’s sand fly fauna and their potential role in disease transmission if any.

## Figures and Tables

**Table 1:** Morphological parameters of female and male *Sergentomyia* (*Neophlebotomus*) *pradeepii* n. sp. (in µm).

Morphological Parameters	Female (N = 10)				Male (N = 10)			
	Max	Min	Mean	SD	Max	Min	Mean	SD
Head length	440	390	412	14	400	360	382	14
Head width	490	440	465	18	440	400	419	14
Interocular distance	190	160	172	11	200	170	182	10
Labrum	270	210	245	18	200	185	196	6
No. of maxillary ventral teeth	5	3	-	-	Rudimentary maxillary teeth			
No. of maxillary lateral teeth	44	40	-	-				
Palpomere length P1	110	90	101	6	90	80	85	4
Palpomere length P2	155	145	149	3	130	120	127	3
Palpomere length P3	160	150	153	3	155	140	150	5
Palpomere length P4	170	155	163	5	175	155	165	7
Palpomere length P5	345	325	338	5	330	310	317	7
Antenna I (f1)	240	220	231	7	270	250	261	6
Antenna II (f2)	115	105	110	4	130	115	123	5
Antenna III (f3)	115	100	109	4	125	115	122	3
Sensilla chaetica in A II	60	55	59	2	55	49	52	3
No. of teeth in cibarium	31	29	-	-	Rudimentary cibarial teeth			
Pharynx length	160	150	155	3	173	160	165	4
Pharynx width	63	55	61	3	58	50	54	3
Pharyngeal armature (depth/length)	45	35	39	3	33	25	29	2
Wing length	1725	1600	1660	47	1650	1500	1570	45
Wing width	650	525	568	37	550	450	508	29
Principal vein length:								
Alpha	540	500	519	15	460	420	438	13
Beta	390	270	302	36	250	220	240	11
Gamma	330	270	297	23	290	250	270	13
Delta	190	160	173	9	180	150	169	10
R5 length	1450	1275	1345	54	1250	1125	1188	40
Fore leg:								
Coxa	325	250	280	26	300	250	283	17
Trochanter	100	75	80	11	75	75	75	0
Femur	800	700	748	30	725	650	680	28
Tibia (T)	775	675	715	32	750	650	705	31
Tarsomeres:								
T1	350	300	328	22	325	275	300	20
T2	225	175	203	18	225	150	188	21
T3	150	125	133	12	150	100	128	14
T4	125	100	113	13	125	100	108	12
T5	100	75	98	8	100	75	83	12
Length of spermatheca	68	58	61	3	-	-	-	-
Width of spermatheca	33	28	29	2	-	-	-	-
Number of segmentations in spermathecal	Spermatheca with striations, not distinct segmentation				-	-	-	-
Length of common spermathecal duct	Common duct not visible				-	-	-	-
Length of spermathecal duct	Spermathecal duct not clear				-	-	-	-
Length of cerci	165	150	157	6	-	-	-	-
Genital furca	Not clearly visible				-	-	-	-
Length of Sperm pump	-	-	-	-	115	100	107	5
Length of Aedeagal duct	-	-	-	-	315	300	310	5
Length of Sperm pump + length of aedeagal duct	-	-	-	-	425	410	417	6
Ratio of length of Aedeagal duct/length of Sperm pump	-	-	-	-	3.1	2.6	2.9	0.1
Length of Paramere	-	-	-	-	185	165	176	6
Length of ejaculatory apodeme	-	-	-	-	90	75	85	5
Length of epandrial lobes	-	-	-	-	230	215	225	5
Gonocoxite length	-	-	-	-	285	265	272	7
Gonostyle length	-	-	-	-	130	110	119	7
Gonostyle spine length	-	-	-	-	95	80	86	5
Length between terminal and sub-terminal spines	-	-	-	-	25	20	23	3
Location of accessory spine from sub-terminal spine	-	-	-	-	Below the sub-terminal spine			

N—number of specimens considered for morphological analysis; Max—maximum; Min—minimum; SD—standard deviation; SC—sensilla chaetica; R-radius; ‘-’—not applicable.

## Data Availability

The datasets generated and/or analyzed during the current study are available from the corresponding author on request.

## Abbreviations

The following abbreviations are used in this manuscript:

COI	Cytochrome c oxidase subunit I
*Neo.*	*Neophlebotomus*
NTD	Neglected tropical disease
SCs	Scheduled Castes
STs	Scheduled Tribes
VL	Visceral leishmaniasis
PKDL	Post Kala-azar Dermal Leishmaniasis
ICZN	International Code of Zoological Nomenclature
gDNA	Genomic DNA
ZSI	Zoological Survey of India
NCBI	National Center for Biotechnology Information
ICMR	Indian Council of Medical Research
VCRC	Vector Control Research Centre
GD	Genetic distance
